# A new *Gymnopus* species with rhizomorphs and its record as nesting material by birds (*Tyrannideae*) in the subtropical cloud forest from eastern Mexico

**DOI:** 10.3897/mycokeys.42.28894

**Published:** 2018-11-21

**Authors:** Enrique César, Victor M. Bandala, Leticia Montoya, Antero Ramos

**Affiliations:** 1 Red Biodiversidad y Sistemática, Instituto de Ecología A.C., P.O. Box 63, Xalapa, Veracruz, 91000, México Instituto de Ecología A.C. Xalapa Mexico

**Keywords:** Marasmioid fungi, Neotropical fungi, nesting biology, Omphalotaceae

## Abstract

A new species of *Gymnopus* is described on the basis of collections from the subtropical cloud forest of eastern Mexico. Macro- and micromorphological characters, in combination with ITS sequences obtained from fruit body tissues, were used for its taxonomic circumscription. Basidiomata of this species were found growing scattered on fallen twigs of *Quercus* and also developing abundant long, black, wiry rhizomorphs. The authors discovered that these latter are used as part of nesting material by *Myonectesoleaginous* (Tyrannidae) inhabiting the subtropical cloud forest studied. A macro- and microscopical description as well as a discussion and illustrations are provided. A new combination in *Gymnopus* is proposed for *Marasmiuswestii*, a synonym of *Marasmiusbrevipes*.

## Introduction

The Santuario de Bosque de Niebla (SBN) is a secondary-growth subtropical cloud forest, persisting as the main peri-urban natural forested area (ca. 30 ha) at southwest Xalapa City, Veracruz (east coast of Mexico). Part of it was a shade-grown coffee plantation abandoned several years ago and nowadays, the SBN (formerly called Parque Ecológico Francisco Javier Clavijero) is a forest ecosystem whose canopy is dominated mostly by trees of *Quercus*, *Carpinus*, *Clethra*, *Oreopanax*, *Ostrya* and *Turpinia*, amongst others. It is an area under conservation and protection by the Instituto de Ecología A.C. and the forest is functioning as an important refuge and reservoir of biological diversity. Permanent systematic field observations carried out on the site are allowing us to document the macrofungal community with special attention to the diversity and ecology of mushrooms (agarics, boletes and milk caps) and it has given us the opportunity to discover new or unusual species of different taxonomic groups, for example *Crepidotus*, *Crinipellis*, *Hygrocybe*, *Lactarius* and *Lepiota* (Bandala and Montoya 2004; Bandala et al. 2006, 2008, 2012, 2016; Montoya and Bandala 2004, 2005, 2008; Montoya et al. 2005).

In the present study, specimens of a marasmioid species producing small basidiomata and abundant black, wiry rhizomorphs were found growing scattered on fallen twigs of *Quercus*. Macro- and microscopical features of basidiomata (hyaline basidiospores, pileipellis non-gelatinous of repent hyphae with diverticulate terminal elements; glabrous, central stipe with homogeneous trama of unbranched hyphae; well-developed rhizomorphs) suggested that our samples relate to members of Marasmiussect.Androsacei Kühner (Desjardin 1987; Desjardin and Petersen 1989). With the advance of molecular systematics on this and other taxonomic groups of marasmioid or even gymnopoid fungi, evidence has been obtained by different authors to recognise that several species earlier placed in sections within the genera *Marasmius* Fr., *Marasmiellus* Murrill and *Micromphale* Gray have phylogenetic relationships with members of *Gymnopus* (Pers.) Gray (Moncalvo et al. 2002; Wilson and Desjardin 2005; Mata et al. 2007; Petersen and Hughes 2016; Tkalčec and Mešić 2013; Antonín and Noordeloos 2010). Members of section Androsacei within *Gymnopus*, for example, show close relationships with the species of Micromphalesect.Perforantia Singer and sect. Rhizomorphigena Singer (Moncalvo et al. 2002; Mata et al. 2004; Wilson and Desjardin 2005¸ Mata et al. 2007; Petersen and Hughes 2016).

A phylogeny, based on ITS sequences obtained here from basidiomata and rhizomorphs collected in the study area, including sequences (downloaded from GenBank: https://www.ncbi.nlm.nih.gov/genbank/) of related marasmioid/gymnopoid fungi, revealed indeed, the phylogenetic relationships of the Mexican species within *Gymnopus*. The macro- and micro-morphological features depicted in this fungus, as well as its distinct position in the phylogenetic analysis, allowed its recognition as a new species which is proposed here. A description accompanied of photographs of basidiomata, illustrations of microscopic features, the displayed phylogeny on the basis of ITS sequences and a taxonomic discussion are provided in this article. During the course of samplings of the *Gymnopus* species studied, we discovered that the long, wiry black rhizomorphs occur in fallen twigs or entangled in aerial branches in the low canopy level, where they are available for use as nesting material by bird species of the Tyrannidae that inhabit the forest under study, which is also discussed.

## Materials and methods

### Sampling and morphological study

Between May 2016 and June 2017, weekly explorations were conducted in the Santuario del Bosque de Niebla, Instituto de Ecología, A.C., at Xalapa. Fresh basidiomata and their rhizomorphs were gathered on fallen twigs of *Quercus*. Some rhizomorphs were also collected from aerial tree branches at, or a little higher, than breast height and others directly from bird nests hanging from branches of a tree of *Turpiniainsignis* (H.B. & K.) Tul. Descriptions of macroscopic characters are based on fresh collections which were photographed and their colours recorded following Kornerup and Wanscher (1967) and Munsell (1994). Microscopic observations were made on dried material mounted in potassium hydroxide (KOH) 3% and stained with 1% Congo red or analszed in Melzer´s solution (Largent et al. 1977). Thirty-five basidiospores per collection were measured in length and width, following the protocol of Bandala et al. (2012). Symbols *x̄_m_* and *q̄_m_* in descriptions refer to the range of mean values per collection (n = 4 collections) of length and width and length/width ratio of basidiospores in side view, respectively. Line drawings were made using a drawing tube. Collections are part of XAL herbarium (Thiers 2018).

### DNA extraction, PCR amplification and sequencing

**Figure 1.**
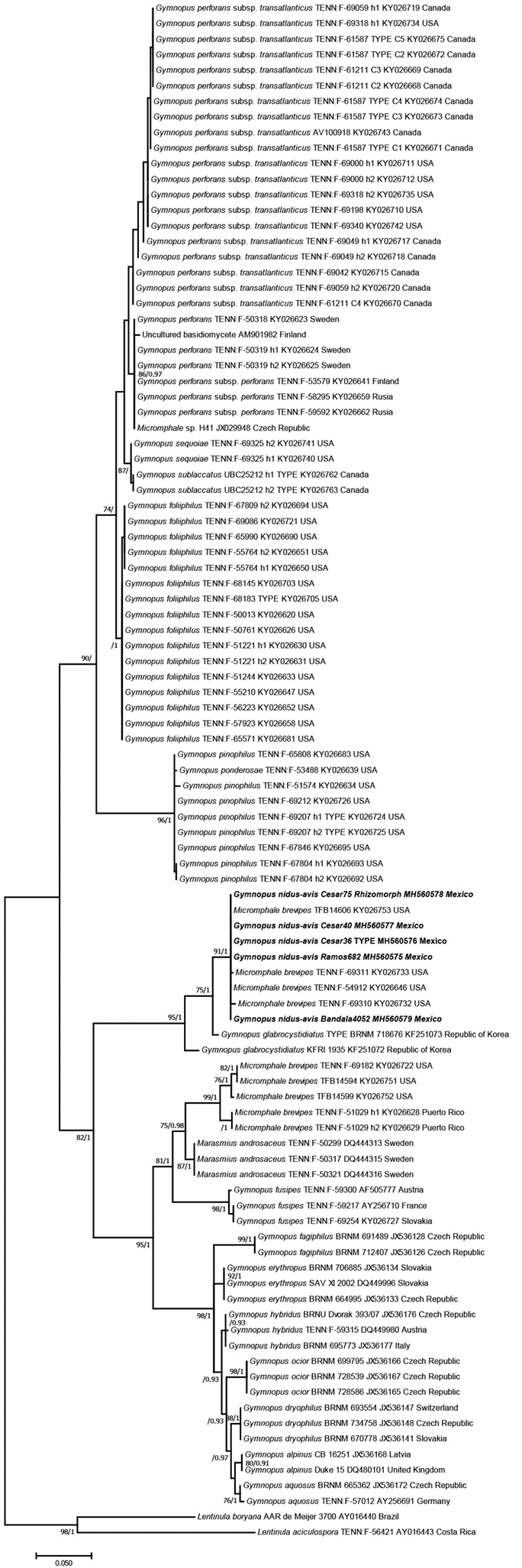
Phylogenetic relationships within *Gymnopus* species inferred from the ITS sequence dataset by maximum likelihood method (ML). Tree with the highest log likelihood (-3619.93). Initial tree(s) for the heuristic search were obtained automatically by applying Neighbour-Joining and BioNJ algorithms to a matrix of pairwise distances estimated using the Maximum Composite Likelihood (MCL) approach and then selecting the topology with superior log likelihood value. The bootstrap values and Bayesian posterior probabilities (obtained after Bayesian inference) are indicated on the tree branches (BS/BPP). Sequences obtained in this study are in bold.

The extraction of genomic DNA of basidiomes and rhizomorphs was performed using the DNA kit extraction Exgene Plant SV mini (GeneAll Biotechnology, Co). PCR was performed to amplify the ITS (Internal Transcribed Spacer) using primers ITS1F, ITS5/ITS4, (White et al. 1990; Gardes and Bruns 1993). PCR conditions: (i) initial denaturation at 95 °C for 5 min; (ii) 35 cycles of 30 sec at 95 °C, 30 sec at 55 °C and 40 sec at 72 °C; and (iii) a 5 min final elongation at 72 °C. Amplified PCR products were sequenced (Macrogen Inc., Seoul, Korea) using a Genetic Analyzer 3730XL (Applied Biosystems). Once sequences were assembled and edited, they were deposited at GenBank database (Benson et al. 2017) with accession numbers indicated in Fig. 1.

### Phylogenetic methods

A dataset, using PhyDE v.0.9971 (Müller et al. 2010), was constructed with the sequences obtained in this study together with related sequences retrieved from GenBank database (http://www.ncbi.nlm.nih.gov) identified with the aid of BLAST tool. The dataset was complemented with other available ITS sequences of *Gymnopus* species at GenBank (Fig. 1), representing the sections *Androsacei*, *Gymnopus*, *Levipedes* (Fr.) Halling, *Perforantia* and *Rhizomorphigena* (after Antonín et al. 2014; Petersen and Hughes 2016). The evolutionary model that best fitted the data and a phylogenetic analysis, under maximum likelihood (ML) (500 bootstrap replications) were achieved with MEGA 7.0 (Kumar et al. 2016), while a phylogenetic analysis under Bayesian Inference (BI) with MrBayes v 3.2.6 (Ronquist et al. 2012; Montoya et al. 2014). *Lentinula* species were included as outgroup taxa (Fig. 1; alignment in TreeBASE 22984). Resulting phylogenetic trees were displayed using Mega 7.0 and FigTree v1.4.3 (Rambaut 2016), respectively. Only bootstrap values (BS) of ≥70% and Bayesian posterior probabilities (BPP) of ≥0.90 were considered and indicated on the tree branches (BS/BPP) of Fig. 1.

## Results

We recovered four fresh collections of basidiomata from which four ITS sequences were generated, including one from a rhizomorph (Fig. 1). In the inferred molecular phylogeny, that included 95 sequences of marasmioid/gymnopoid taxa worldwide (Fig. 1), the five generated sequences of the Mexican *Gymnopus* species clustered in a strongly supported and isolated clade (91/1.0), sister to *G.glabrocystidiatus* Antonín, R. Ryoo & K.H. Ka known from the Republic of Korea (Antonín et al. 2014). Both species in the analysis appear together in a well-supported branch (95/1.0), separated from other sequences (after Petersen and Hughes 2016) supporting phenotypically lookalike marasmioid species as *Micromphalebrevipes* (Berk. & Ravenel) Singer traditionally placed in sect. Rhizomorphigena (clade with high support 99/1.0) or *Gymnopusandrosaceus* (L.) J.L. Mata & R.H. Petersen from sect. Androsacei (clade with high support 87/1.0) (Fig. 1), which probably suggests that Mexican and Korean taxa belong to a different section or, in concordance with other authors, the resultant clades reflect the relationships amongst closely related taxa of *G.androsaceus* complex (Wilson and Desjardin 2005; Mata et al. 2007; Hughes and Petersen 2016). Taking into account both the results of the phylogenetic analysis and the distinctive set of morphological features that the Mexican *Gymnopus* specimens possess (see description below), we concluded that they represent a new *Gymnopus* species which inhabits the subtropical cloud forest from eastern Mexico and it is proposed here. In the discussion below, we comment on the specimens supporting the sequences recorded as “*Micromphalebrevipes*”, some of which appear in the phylogeny nested with the Mexican taxon, while others clustered in a separate clade (Fig. 1).

### Gymnopus nidus-avis

Taxon classificationFungiAgaricalesOmphalotaceae

César, Bandala & Montoya sp. nov.

MB827326

Figs 2 , 3 , 4

#### Holotype.

MEXICO. Veracruz: Municipality of Xalapa, Santuario del Bosque de Niebla, Instituto de Ecología A.C., 1343 m a.s.l., gregarious, on fallen twigs of *Quercus*, 20 April 2016, Cesar 36 (XAL).

#### Diagnosis.

Pileus pale brown to brown. Lamellae adnexed, distant, very pale brown. Basidiospores ellipsoid to subcylindrical. Basidia 2–4-spored, narrowly clavate. Cheilocystidia 20–39 × 3–8 µm, irregularly cylindrical, with constrictions and small lateral appendages. Pileipellis hyphae with colourless incrustations; terminal elements appendiculate. Pileus and lamellar tissues clampless.

**Figure 2.**
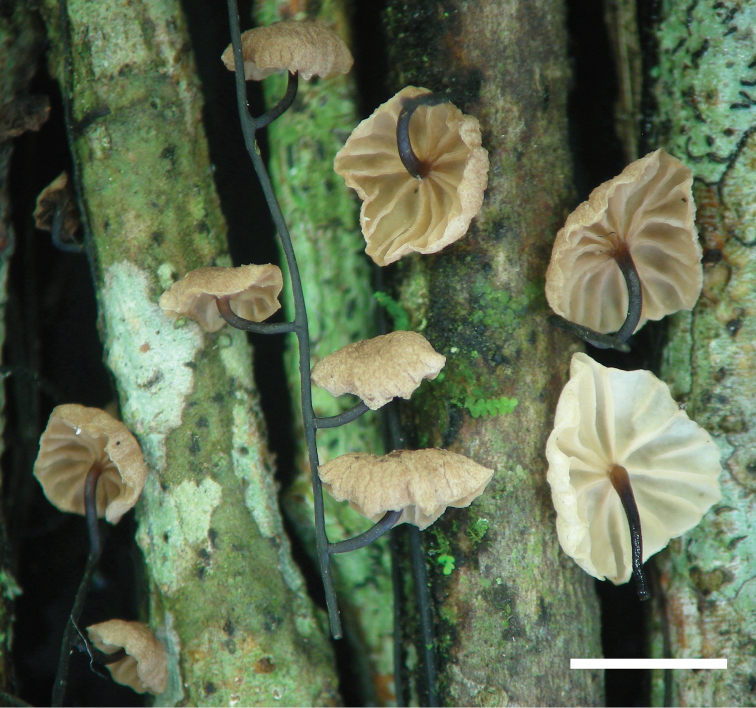
*Gymnopusnidus-avis*: basidiomata (César 36, holotype). Scale bar: 4 mm

#### Gene sequences ex-holotype.

MH560576 (ITS).

#### Etymology.

Referring to the use of rhizomorphs as nesting material by birds.

*Basidiomata* marcescent. *Pileus* 1–7 mm diam., convex to plano-convex, usually somewhat depressed over the disc and occasionally slightly infundibuliform with age, some developing a weak umbo, smooth or weakly rugulose, subtly striate when young becoming somewhat sulcate and then wavy towards the margin, this latter slightly decurved, surface dry, minutely granulose under lens, matt, pale brown (7.5 YR 7/4–6; 10YR 7/4) to brown (7.5 YR 5/6; 10 YR 4/4); context thin (up 1 mm thick), soft, whitish. *Lamellae* adnexed, distant (7–17), very pale brown (2.5 Y 8/2), narrow to moderately broad (up to 1 mm broad), sometimes forked, lamellulae of two different lengths, rarely weakly intervenose with age, margin entire. *Stipe* 1–12 × 0.2–0.3 mm, central, sometimes only slightly eccentric, cylindrical or tapered towards the base, straight, often curved, solid, glabrous, very finely striate (under lens), reddish-brown at the apex (2.5 YR 4/6), dark brown to black below (10YR 2/1, 7.5YR 2.5/2), insititious, at times erumpent, arising either from the substratum or from rhizomorphs; context light brown (2.5Y 6/4). *Rhizomorphs* up to 500 × 1 mm, simple, black, wiry, abundant. Odour and taste not distinctive.

*Basidiospores* 7–10 × 3–5.5 µm, *x̄_m_* = 8.3–9.1 × 3.7–4; *q̄_m_* = 2–2.4 (*n* = 4), ellipsoid to subcylindrical, somewhat lacrymoid with a weak suprahilar depression, hyaline, inamyloid, thin-walled. *Basidia* 20–41 × 5–10 µm, 2–4-spored, clavate to narrowly clavate, hyaline, inamyloid, thin-walled, clampless. *Cheilocystidia* 20–39 × 3–8 µm, irregularly cylindrical to narrowly-claviform, simple or usually bifurcate, with small lateral appendages and constrictions, moderately abundant, shortly projected beyond the hymenium level, hyaline, inamyloid, thin-walled, clampless. *Pilleipellis* composed of compactly interwoven, cylindrical, non-gelatinised, thin-walled, clampless, hyaline hyphae, 4–8 µm diam., irregularly covered by colourless, refractive incrustations of fine or moderately broad, discontinuous lines, arranged in a more or less irregularly transversal pattern, bearing repent or slightly erect, hyaline, slightly dextrinoid terminal elements which are irregularly cylindrical, with numerous appendages or with short to moderately large lateral outgrowths, thin-walled or the apices often thick-walled, with a morphology similar to a Rameales-structure. *Pileus trama* hyphae interwoven, 4–6 μm diam., cylindrical, often bifurcate, thin-walled, hyaline, weakly dextrinoid, smooth, often intermixed, some covered with colourless refractive encrusting material. *Hymenophoral trama* regular to subregular, with cylindrical, thin-walled, hyaline, inamyloid to weakly dextrinoid, clampless hyphae 3–5 μm diam. *Stipitipellis* composed of repent, cylindrical, thick-walled, heavily dark brown pigment-encrusted, clampless hyphae 5–6 μm diam., dextrinoid; with scattered, hyaline or brown-pigmented, diverticulate terminal elements 4–19 (–21) × 3–4 (–5) µm, thin-walled. *Stipe trama* hyphae more or less parallel, composed by cylindrical or more or less ventricose hyphae, 4–15 (–20) μm diam., thick-walled (1–5 μm thick), smooth or with colourless encrusting material, towards the central medulla they appear intermixed with hyaline, smooth, cylindrical hyphae, 3–6 µm diam., thin or slightly thick-walled (<1 µm thick), occasionally clamped. *Clamp connections* absent in pileus and lamellar tissues, present in the slender, medullary hyphae of stipe.

**Figure 3.**
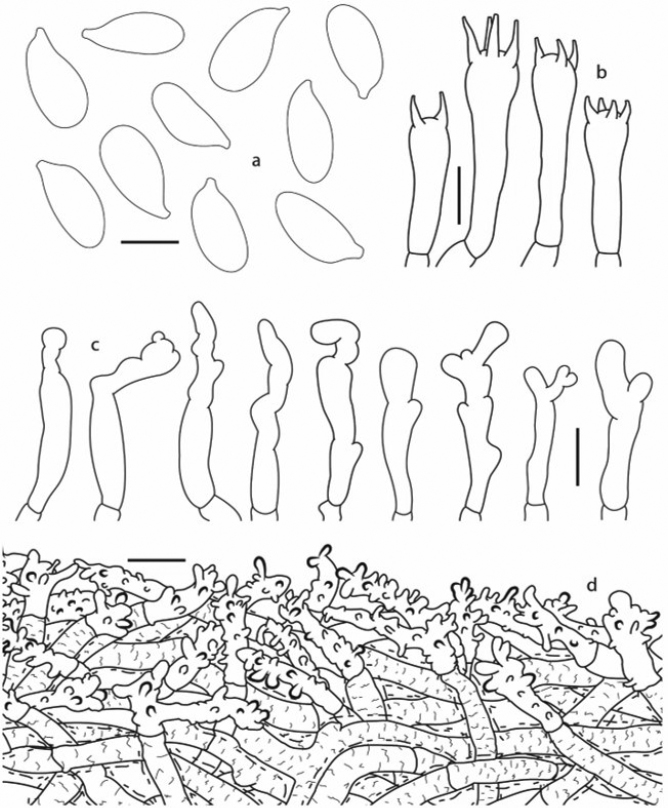
*Gymnopusnidus-avis*: **a** Spores **b** Basidia **c** Cheilocystidia **d** Pileipellis (holotype). Scale bars: 5 µm (**a**); 10 µm (**b–d**).

#### Habitat.

In subtropical cloud forest, scattered or gregarious on fallen twigs of *Quercus*, often the basidiomes arising directly from the wiry, black rhizomorphs and these latter at times are entangled, hanging from aerial branches.

#### Additional specimens examined.

MEXICO. Veracruz, Municipality of Xalapa, Santuario del Bosque de Niebla, Instituto de Ecología A.C., 1343 m a.s.l., 18 May 2006, Bandala 4052; 7 July 2016, César 41; 10 Aug 2016, Ramos 682 (all at XAL).

**Figure 4.**
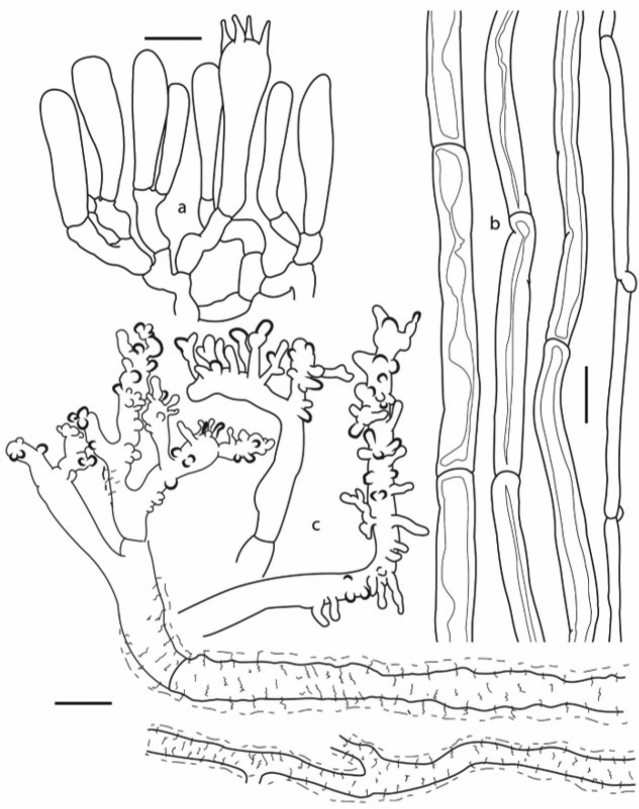
*Gymnopusnidus-avis*: **a** Hymenial trama elements **b** Thick- and thin-walled hyphae from stipe medullary tissue **c** Hyphae and terminal elements of pileipellis (holotype). Scale bars: 10 µm (**a–c**).

## Discussion

Amongst the species that produce tiny, marcescent basidiomes and long, black, wiry rhizomorphs, *Gymnopusnidus-avis* can be recognised by the colour of pileus and lamellae, these latter adnexed and distant, size and shape of basidiospores and cheilocystidia, 2–4-spored basidia, pilleipellis hyphae bearing colourless, refractive encrusting material, with appendiculate terminal elements (similar to a Rameales-structure) and with the pileus hyphae and lamellar trama (hymenial elements included) lacking clamp connections. Interestingly, the presence of clamp connections exclusively is confined to the slender, thin-walled, hyphae of stipe trama, even mycelia obtained from tissues in axenic culture did not present clamped septa. The Mexican species is genetically close to the Korean *G.glabrocystidiatus*, with which it shares morphological features as the filiform stipe, pileipellis composed of encrusted, diverticulate hyphae and clampless hyphae. Basidomata of *G.glabrocystidiatus*, however, are slender and longer (pileus 4–8 mm; stipe 15–40 × 0.5 mm) lacking rhizomorphs, grow on needles of *Abies* and have broadly clavate or pyriform cheilocystida, 2-spored basidia and terminal elements of the pileipellis with irregular coralloid shape or broom-like (Antonín et al. 2014).

The new species is macro-morphologically similar to *Marasmiusbrevipes* Berkeley & Ravenel (*Micromphale*, Singer, in Dennis 1953), a species occurring in southern USA, growing also on *Quercus* sticks, with other known records from Alaska, Martinique, Puerto Rico and Trinidad (Dennis 1953; Pegler 1983; Desjardin and Petersen 1989; Petersen and Hughes 2016). The differences between both species are very subtle in the pileus and lamellae colours, being in *M.brevipes* brown or dark reddish-brown, with a slightly darker disc and brownish-grey to light brown or pale pinkish-cinnamon colours, respectively. However, the pileus of *M.brevipes* is more markedly striate, even sulcate, plane at centre (i.e. without evidence of umbo), besides having adnate lamellae and shorter stipe (1–2.5 × <0.5 mm; 1–4 × 1 mm or 4–7 × 0.2–0.4 mm) which is often eccentric (Dennis 1951, 1953; Pegler 1983; Desjardin and Petersen 1989). Microscopically *M.brevipes* is a very distinctive species that may be readily recognised by the heavily brown pigment-encrusted pileipellis elements, with short, coralloid terminal hyphae, thick-walled, hyaline or pale brownish, smooth or weakly encrusted, inamyloid hyphae of pileus and lamellar trama, clamp connections common on all tissues and amygdaliform and less cylindrical basidiospores (Dennis 1951, 1953; Desjardin and Petersen 1989), in contrast with those of *Gymnopusnidus-avis*.

Results of the phylogenetic analysis (Fig. 1) suggest that *G.nidus-avis* and *Marasmiusbrevipes*, both with small, tiny basidiomata and long black rhizomorphs, to some extent could be easily confused. Several sequences treated by Petersen and Hughes (2016) under “*Micromphalebrevipes*” were included in the present study (Fig. 1), three of them grouping in the same clade together with the sequences of the Mexican specimens. Staff at TENN Herbarium confirmed to us that two of these specimens (TENN 54912 and 69310) have clampless hyphae in pileus and lamellar tramae, hence these specimens are interpreted here to be contaxic with the Mexican species and not with the type specimen of *Marasmiusbrevipes* which possesses clamp connections on all tissues, including the basidia, cheilocystidia and pileipellis elements, as described by Desjardin and Petersen (1989) and in the type study of *Marasmiusbrevipes* by Desjardin (1989). Other sequences labelled also under “*Micromphalebrevipes*”, in the phylogeny inferred (Fig. 1) appeared in a separate clade with high support (99/1.0). They belong to samples TENN 51029 and 69182 which have clamp connections in pileus and lamellar trama, suggesting that some of them could be contaxic with the type specimen of *Marasmiusbrevipes* representing the *Rhizomorphigena* section (Hughes and Petersen 2016).

*Marasmiusbrevipes* is a species accepted and validly published (Berkeley and Curtis 1853; Desjardin and Petersen 1989). If the species is recognised to belong to the group of marasmioid species phylogenetically close to Gymnopussect.Androsacei (Kühner) Antonín & Noordel., as suggested by the analyses obtained by Hughes and Petersen (2016) and here (Fig. 1), we note that the species has not been transferred to *Gymnopus*. The name *Gymnopusbrevipes* (Bull.) Gray, however, is occupied by an accepted synonym for *Melanoleucabrevipes* (Bull.) Pat. (Index Fungorum; Mycobank 486476). An alternative name is that of the synonym, *Marasmiuswestii* Murr., following a type study by Desjardin (1989) that documented the presence of clamp connections (see also Desjardin and Petersen 1989 and Hesler 1959). The new combination for that marasmioid species seems to be pertinent (Arts. 6, 41) and the following is proposed:

### Gymnopus westii

Taxon classificationFungiAgaricalesOmphalotaceae

(Murrill) César, Bandala & Montoya comb. nov.

MB828158

#### Basionym.

*Marasmiuswestii* Murrill, Proc. Florida Acad. Sci. 7:110. 1945.

Syn.: *Marasmiusbrevipes* Berk. & Ravenel, in Berkeley and Curtis, Ann. Mag. Nat. Hist., Ser. 2 12: 426. 1853.

=*Micromphalebrevipes* (Berk. & Ravenel) Singer, in Dennis, Kew Bull. 8: 42. 1953.

Not *Agaricusbrevipes* Bull., Herb. Fr. 11: tab. 521. 1791 (*Gymnopus*, Gray, Nat. Arr. Brit. Pl. 1: 609.1821; *Melanoleuca*, Pat., Essai Tax. Hyménomyc.: 158, 1900.).

Reports of marasmioid fungi as nesting material for Passeriformes have been referred in several works as filaments, rhizomorphs or horse-hair fungi and recorded from the Nearctic and the Neotropical regions (Sick 1957; Mc Farland and Rimmer 1996; Aubrecht et al. 2013). These fungal materials have been identified as *Marasmiusandrosaceus, M.brevipes, M.crinis-equi* F. Muell. ex Kalchbr., *M.nigrobrunneus* (Pat.) Sacc. and *M.* sp. Fungal material from *Marasmius* sp. and *Crinipellis* sp. was recorded in Mexico as being associated with nests of birds in a tropical forest from Tabasco in the south of Mexico (Gómez et al. 2014). In the present study, one of the sequences (MH560578), included in the obtained phylogeny (Fig. 1), belongs to a rhizomorph of *Gymnopusnidus-avis* re-collected in a nest of *Myonectesoleaginous* Lichtenstein. This *Gymnopus* species represents a new species in the list of marasmioid taxa found interacting with birds.

All the basidiomes collected in the present study were found on fallen twigs in the low canopy level but it is possible that fructifications occur also on rhizomorphs at the top of the trees, where these latter are found and used by birds. Previous reports have suggested that bird efforts of picking this inconspicuous material is rewarded with the high tensile strength, reduced water uptake and antimicrobial properties of the rhizomorphs, which consequently protect the offspring (Aubrecht et al. 2013; Freymann 2007). Preliminary results, based on various sequences obtained from rhizomorphs gathered in different nests of bird species found in the study site, suggest the presence of an important diversity of marasmioid rhizomorph-forming species in the cloud forest studied. It is interesting to note also that we could evidence the presence of nests of wasps of *Polybiarejecta* Fabricius, near one of *Myonectesoleagineus* built with rhizomorphs of *Gymnopus*. It coincides with the observations made by Joyce (1993) in Costa Rica regarding the presence of nests of that wasp species near *Tolmomyassulphurescens* and *Cacicus* spp. nests. These latter authors concluded that such association could reduce predation, remarking the importance of the fungal rhizomorphs in this complex ecological interaction.

## Supplementary Material

XML Treatment for Gymnopus nidus-avis

XML Treatment for Gymnopus westii
